# Supplementary figures and images for: Identification of miRNA–mRNA Networks Associated with Pigeon Skeletal Muscle Development and Growth
Source: Animals (Basel). 2022 Sep 21;12(19):2509. doi: 10.3390/ani12192509 (PMC9558527; doi:10.3390/ani12192509)

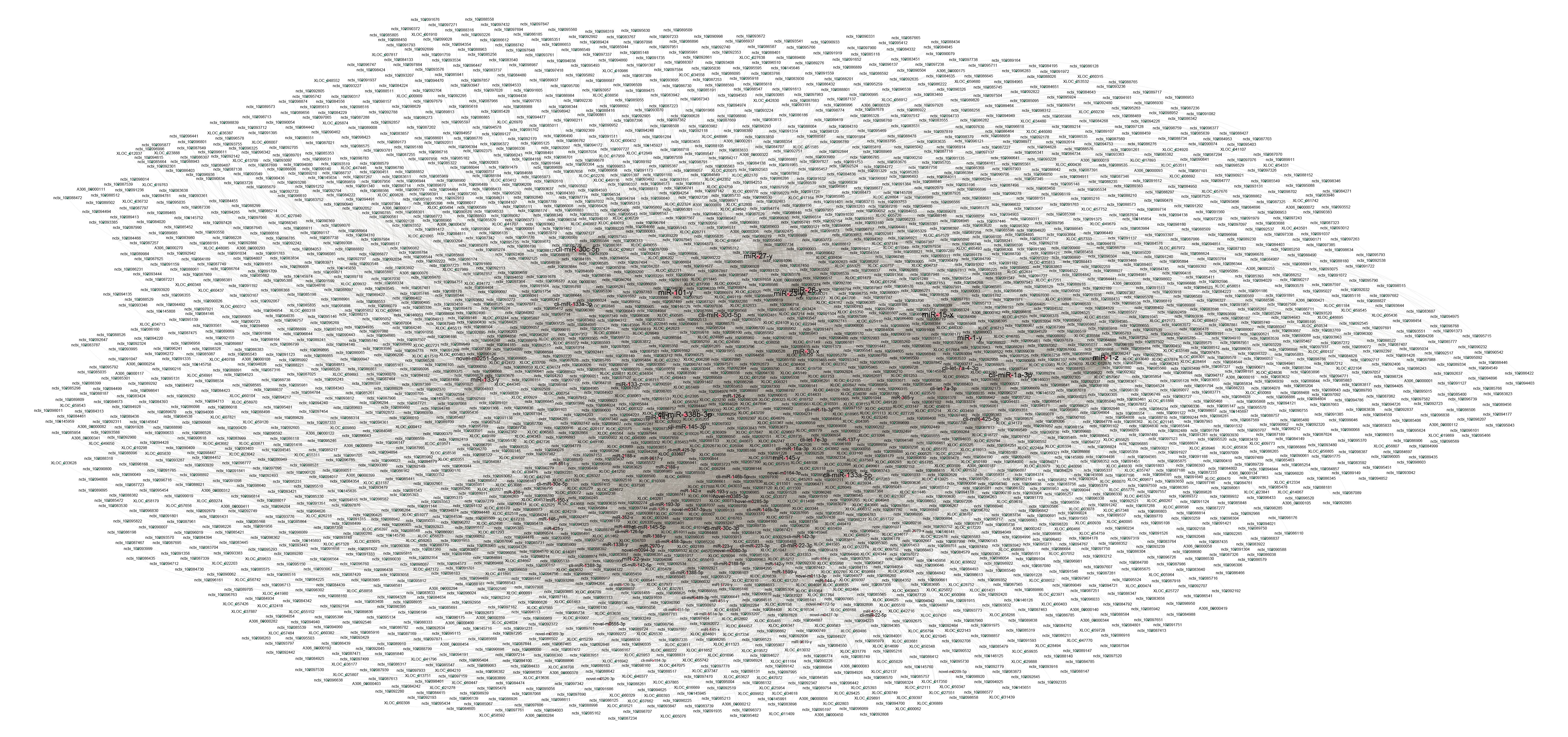

Supplement: Supplementary file 1 [file animals-12-02509-s001.zip › Figure S2.tif]
